# Creation of Triple Hierarchical Micro-Meso-Macroporous N-doped Carbon Shells with Hollow Cores Toward the Electrocatalytic Oxygen Reduction Reaction

**DOI:** 10.1007/s40820-017-0157-1

**Published:** 2017-09-27

**Authors:** Ruohao Xing, Tingsheng Zhou, Yao Zhou, Ruguang Ma, Qian Liu, Jun Luo, Jiacheng Wang

**Affiliations:** 1 School of Materials Science and Engineering, Shanghai University, 99 Shangda Road, Shanghai, 200444 People’s Republic of China; 2 State Key Laboratory of High Performance Ceramics and Superfine Microstructure, Shanghai Institute of Ceramics, Chinese Academy of Sciences, 1295 Dingxi Road, Shanghai, 200050 People’s Republic of China; 3 Shanghai Institute of Materials Genome, Shanghai, People’s Republic of China

**Keywords:** Hierarchical pores, Hollow cores, N doping, Electrocatalysis, Oxygen reduction reaction

## Abstract

A series of triple hierarchical micro-meso-macroporous N-doped carbon shells with hollow cores have been successfully prepared via etching N-doped hollow carbon spheres with CO_2_ at high temperatures. The surface areas, total pore volumes and micropore percentages of the CO_2_-activated samples evidently increase with increasing activation temperature from 800 to 950 °C, while the N contents show a contrary trend from 7.6 to 3.8 at%. The pyridinic and graphitic nitrogen groups are dominant among various N-containing groups in the samples. The 950 °C-activated sample (CANHCS-950) has the largest surface area (2072 m^2^ g^−1^), pore volume (1.96 cm^3^ g^−1^), hierarchical micro-mesopore distributions (1.2, 2.6 and 6.2 nm), hollow macropore cores (~91 nm) and highest relative content of pyridinic and graphitic N groups. This triple micro-meso-macropore system could synergistically enhance the activity because macropores could store up the reactant, mesopores could reduce the transport resistance of the reactants to the active sites, and micropores could be in favor of the accumulation of ions. Therefore, the CANHCS-950 with optimized structure shows the optimal and comparable oxygen reduction reaction (ORR) activity but superior methanol tolerance and long-term durability to commercial Pt/C with a 4e^−^-dominant transfer pathway in alkaline media. These excellent properties in combination with good stability and recyclability make CANHCSs among the most promising metal-free ORR electrocatalysts reported so far in practical applications.

**Electronic supplementary material:**

The online version of this article (doi:10.1007/s40820-017-0157-1) contains supplementary material, which is available to authorized users.

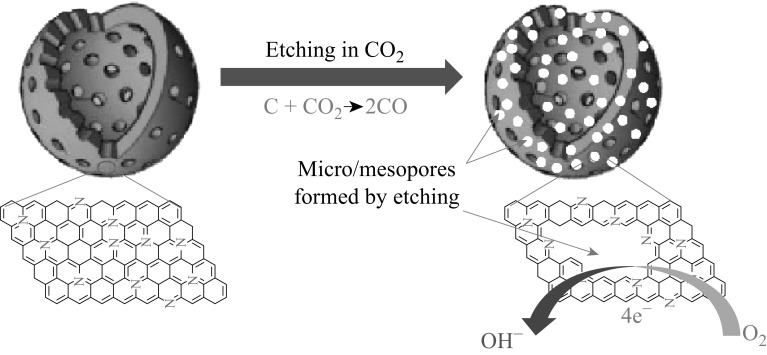

## Highlights

- A series of triple hierarchical micro-meso-macroporous N-doped carbon shells have been successfully prepared via etching N-doped hollow carbon spheres with CO_2_ at high temperatures.
- The activated sample has the large surface area and pore volume, hierarchical micro-mesopore distributions, hollow macropore cores and controllable nitrogen contents.
- The optimized sample shows the comparable oxygen reduction reaction activity but superior methanol tolerance and long-term durability to commercial Pt/C with a 4e^−^-dominant transfer pathway in alkaline media.

## Introduction

Fuel cells, which serve as a highly efficient, environment-friendly energy transformation system, have received intensive research and focus in transports, aerospace and communication equipment during these years [1–3]. However, the wide applications of fuel cells remain a great challenge because of the slower rate of the cathodic oxygen reduction reaction (ORR) [4, 5]. For the ORR process, it encounters difficulties in absorbing O_2_ and breaking O–O double bonds in the cathode. Thus, an infinite energy barrier of the ORR has become one of the most important factors limiting the performance of fuel cells [3, 6–8]. So far, Pt-based materials have been regarded as one of the most active catalysts for the ORR and they can be commercially available at present [9–12]. Pt-based catalysts are highly effective, but they are subjected to some drawbacks including high cost, limited resources and the crossover to methanol. Thus, the large-scale production and commercialization of these Pt-based catalysts in fuel cells are severely limited [13, 14]. So, there is therefore an urgent need, but it is still a significant challenge to reduce the cost and enhance the stability for the ORR catalyst via the design and preparation of non-precious metal ORR electrocatalysts.

A series of nanostructured carbon-based nanostructured materials, such as carbon nanotubes (CNTs) [15, 16], carbon spheres [17, 18], mesoporous carbon [19, 20] and carbon quantum dots [21], have been explored in varied applications including supercapacitors [22–24], gas adsorption [25, 26], removal of heavy metal ions [27], biomedical imaging [28], in virtue of their high stability, low cost and easy availability. The doped heteroatoms (e.g., boron, nitrogen, phosphorus, sulfur and fluorine) with distinct electronegativity with respect to carbon atoms cause the electron modulation to change the charge distribution, electronic properties and chemical activities of the doped carbons [29–31]. Doping carbon materials with nitrogen, which has a similar atomic size to carbon but different electron configuration, is highly effective to improve the ORR activity by adjusting the electronic structures and electron distribution [32]. There are some excellent reviews in the literature dealing with the N-doped carbon materials [33–36]. Recently, various N-doped carbon nanomaterials, including graphene sheets [13, 37–40], CNTs [41–45], carbon nanofibers [12, 46], biomass-derived carbon [47], small molecule precursors [48] and hollow carbon spheres (HCSs) [17, 49–52] have been investigated as the metal-free ORR electrocatalysts due to their unique electronic properties derived from the conjugation between the nitrogen lone-pair electrons and the graphene π system [31, 53–56]. However, there is still a gap between heteroatom-doped carbon materials and commercial Pt/C. Thus, the continuous searching for highly active metal-free ORR electrocatalysts still remains challenging.

There is no denying that the construction of a nanostructured catalyst with hierarchical porosity and large surface area is beneficial for improving the activity thanking to more accessible active sites [57], fast diffusion of reactants to the active sites and fast release of the products from the sites. The activated porous carbons possess a highly developed porosity with adjustable and hierarchical structure which can be used in a wide range of fields, such as gas- or liquid-phase adsorption processes and electrochemical energy storage [58, 59]. In the field of electrochemical catalysis, the large surface area and porosity could improve the catalytic activity by enlarging the limiting current density and lowering the onset potential. CO_2_ activation is an efficient strategy to increase the porosity and adjust the pore structures of various carbon materials by etching carbon with CO_2_ at high temperatures. The method of CO_2_ activation has been applied in various carbon-based materials including resorcinol–formaldehyde resin [60], carbon nanofibers [46] and CMK-1 [61].

In this work, we successfully developed a kind of novel porous N-doped carbons shells with large hollow cores and triple micro-meso-macroporosity (1.1, 2.6, 6.2, ~91 nm) by etching N-doped hollow carbon spheres (NHCSs) with CO_2_ at high temperatures. The CO_2_ activation treatment allows the formation large amount of micropores and narrow mesopores within the shells, thus significantly improving the surface area and pore volume, although the total N contents show a downward trend from 7.6 to 3.8 at% with increasing the activation temperature from 800 to 950 °C. In the activated samples, the pyridinic and graphitic nitrogen groups are dominant among various N-containing groups, which are active for the ORR. Among various samples, the 950 °C-activated sample (CANHCS-950) has the largest surface area (2072 m^2^ g^−1^) and pore volume (1.96 cm^3^ g^−1^) and highest relative content of pyridinic and graphitic N groups. Due to its unique structures of triple hierarchical micro-meso-macroporosity, large surface area and high-content doping of pyridinic and graphitic N groups, CANHCS-950 demonstrates the best ORR activity among these samples, which is comparable to commercial Pt/C. The macropores of hollow cores (~91 nm) could store up the electrolyte and close the spread distance between the electrolyte and materials. The mesopores (2.6 and 6.2 nm) could reduce the transport resistance of the reactants to the active sites [62–64]. The as-formed micropores could be in favor of the accumulation of ions (1.1 nm) [65]. Moreover, it could catalyze the ORR via a close four-electron reaction process with very low yield of peroxides as the side product, thus obtaining the maximum energy transformation efficacy. Furthermore, it also shows the superior long-term stability and methanol tolerance to commercial Pt/C for ORR, indicating that CANHCS-950 is a good candidate for replacing Pt/C used in fuel cells.

## Experimental Section

### Chemicals

Hexamethylentetramine (HMT), 2,4-dihydroxybenzoic acid (DA), sodium oleate (SO), potassium hydroxide (KOH), anhydrous ethanol (EtOH), carbon dioxide (CO_2_) and ammonia (NH_3_) were purchased from Sinopharm Chemical Reagent Co. Ltd. CH_3_OH was purchased Shanghai Lingfeng Chemical Reagent Co. Ltd. The PEO-PPO-PEO triblock copolymers P123 (EO_20_PO_70_EO_20_, *M*_*v*_ = 5800) and Nafion solution (5 wt%) were purchased from Aldrich. The commercial Pt/C catalyst (20 wt%) was obtained from Johnson Matthey (UK). All solvents and chemicals were utilized as received without any further purification.

### Materials Synthesis

#### Preparation of Hollow Polymer Spheres (HPSs)

The HPSs were prepared via the hydrothermal polymerization as reported by the previous study [26]. Double surfactants P123 and SO were used as the soft templates while DA and HMT acted as the carbon sources. Typically, DA (2.4 mmol) and HMT (2 mmol) were mixed in 120 mL of deionized water under vigorous stirring for at least 10 min until forming a homogeneous solution **A**. SO (0.48 mmol) and P123 (0.015 mmol) were then added into 40 mL deionized water under stirring for about 60 min to obtain homogeneous solution **B**. Then, solution **B** was dropped into **A** slowly under vigorous stirring. The color of the solution changed gradually from hyaline to milk white. After stirring for 10 min, the mixed solution was transferred into a Teflon-lined stainless steel autoclave with 200 mL and heated at 160 °C for 8 h. After cooling to room temperature, the resulting precipitates (HPSs) were collected by centrifugation and dried at 80 °C under vacuum overnight after washing with deionized water for three times.

#### Preparation of N-doped Hollow Carbon Spheres (NHCSs)

The HPSs were thermally treated at 650 °C for 3 h with a heating rate of 2 °C min^−1^ in NH_3_ atmosphere at a gas flow rate of 40 mL min^−1^ to obtain NHCSs.

#### Activation of NHCSs by CO_2_ at High Temperature

The CO_2_-activated NHCSs (CANHCSs) were prepared by reacting NHCSs with CO_2_ in a tubular furnace at controlled temperatures (800, 900, 950 °C) for 30 min with a heating rate of 10 °C min^−1^. The final products were named as CANHCS-800, CANHCS-900 and CANHCS-950, respectively, depending on the activation temperature.

### Structural Characterization

Scanning electron microscopy (SEM) was carried out using a field emission scanning electron micro-analyzer (FEI Magellan 400), and transmission electron microscopy (TEM) images were taken by JEM-2100F. Raman spectra were collected on a DXR Raman Microscope (Thermal Scientific Co., USA) with 532 nm excitation length. Nitrogen adsorption–desorption isotherms were measured at liquid nitrogen temperature (77 K) with an ASAP 2010 Accelerated Surface Area and Pore Size Analyzer System (Micrometitics, Norcross, GA). During measuring procedures, the samples were treated at 300 °C overnight under vacuum. The specific surface areas were calculated with the Brunauer–Emmett–Teller (BET) method. The total pore volume was calculated from the amount of nitrogen adsorbed at a relative pressure of 0.99. The pore size distribution curves were calculated by means of the desorption branch of the isotherms using the quenched solid density functional theory (QSDFT). X-ray photoelectron spectroscopy (XPS) measurements were taken using an ESCALAB 250 X-ray photoelectron spectrometer using Al *Kα* (*hv* = 1486.6 eV) radiation to analyze the surface of the obtained samples.

### Electrode Preparation and Electrochemical Measurements

The catalyst inks were prepared by mixing 5 mg catalyst with 30 µL of Nafion solution (5 wt%), 500 µL deionized water and 500 µL ethanol under ultrasonic irradiation for 2 h until a uniform mixture was obtained. Then, 20 µL of the ink was dropped on the working electrode by a micro-injector and it was dried at 50 °C for 10 min until the sample formed a film. The Pt/C catalyst (20 wt% Pt on carbon, Sigma-Aldrich) ink was also used for preparing the working electrode in the same way.

The electrochemical measurements were tested in 0.1 M KOH solution using a three-electrode system with an electrochemical workstation (Pine Instrument Co.). A saturated calomel electrode (SCE) and a Pt plate were used as the reference electrode and the counter electrode, respectively. A rotating disk electrode (RDE) was used the working electrode for cyclic voltammetry (CV) or linear sweep voltammetry (LSV) measurements, and a rotating ring-disk electrode (RRDE) was used the working electrode for dual-electrode CV (DECV) measurements. The electrolyte was saturated by using high-purity bubbling N_2_ or O_2_ for 0.5 h before the test, and all the experiments were carried in 25 °C.

In the case of the RDE tests, cyclic voltammetry (CV) measurement was performed from −1 to 0.2 V (vs. SCE) in O_2_-saturated in 0.1 M KOH solution with a sweep rate of 50 mV s^−1^ for 50 times’ cycle for activating electrode and 10 mV s^−1^ for 3 times’ cycle for data recording. Linear sweep voltammetry (LSV) measurements were conducted from 0 to −1.0 V (vs. SCE) in O_2_-saturated with a sweep rate of 10 mV s^−1^ at different rotating speeds of 400, 625, 900, 1225, 1600, and 2025 rpm.

For the RRDE tests, a CG disk (0.2475 cm^2^) was used as a working electrode which was surrounded by a Pt ring (0.1866 cm^2^). The ring potential was held at 0.2 V (vs. SCE) with a rotating speed of 1600 rpm while the scanning rate was 10 mV s^−1^. The ring current and disk current were collected in O_2_-saturated 0.1 M KOH from GC disk and Pt ring, respectively. The apparent number of electrons transferred (*n*) and the percentage of H_2_O_2_ released (%H_2_O_2_) during the ORR process are calculated as follows:

1 $$n = 4\frac{{I_{\text{d}} }}{{I_{\text{d}} + I_{\text{r}} /N}}$$

2 $$\% {\text{H}}_{2} {\text{O}}_{2} = 200\frac{{I_{\text{r}} /N}}{{I_{\text{d}} + I_{\text{r}} /N}}$$

where *I*_d_ is the disk current, *I*_r_ is the ring current and *N* (the value is 0.37) is the collection efficiency of the Pt ring electrode.

## Results and Discussion

**Fig. 1:**
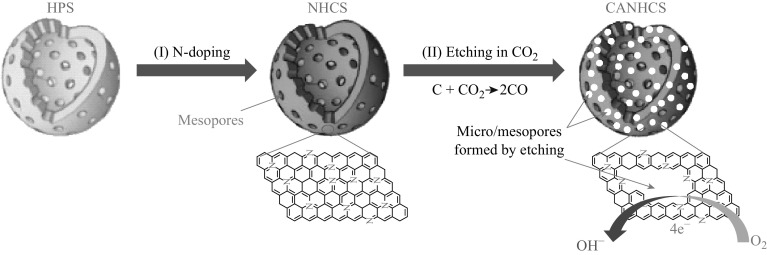
Scheme for the synthesis of CANHCS with hollow macroporous core and hierarchical micro-mesopores as highly active metal-free electrocatalysts with improved activity for the ORR via a direct four-electron reaction pathway. (I) N doping of HPS by thermal treatment in NH_3_ and (II) etching NHCS via the reaction with CO_2_ at high temperature to form micro-mesopores within the shells

**Fig. 2:**
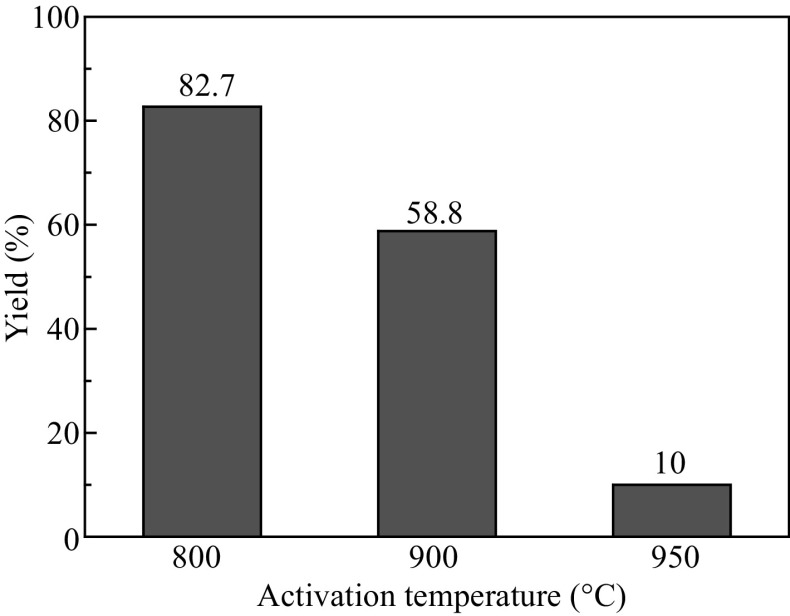
Yield of the activated samples prepared by the activation of NHCSs with CO_2_ at different temperatures. Yield (%) = the ratio of the mass of the activated product divided by the mass of the original weight of NHCSs × 100%

As presented in Fig. 1, NHCSs with hollow macroporous core were synthesized by pyrolysis of HPSs in flowing NH_3_. The subsequent reaction with CO_2_ could generate large amount of additional micro-mesopores within the shells in the CO_2_-activated NHCSs (CANHCSs). This CO_2_ activation process could lead to the significant carbon loss via the release of the volatile matter by the reaction with CO_2_ [60]. The yields of the activated samples at different temperature (800, 900, and 950 °C) could be calculated as follows:

3 $${\text{Yield }}\left( \% \right) \, = \, \left[ {W_{1} /W_{2} } \right] \, \times 100\%$$

where *W*_1_ is the weight of CANHCSs and *W*_2_ is the weight of parent NHCSs. As expected, the yield evidently decreases with the increase in the activation temperature, implying that more carbon burns off at higher temperature via the reaction with CO_2_ (Fig. 2). At 800 °C, the carbon yield is 82.7% and the 900 °C activation results in the lower carbon yield (58.8%). At 950 °C, only 10.0% carbon was remained, implying the majority of carbon burned away. These results and trends are well consistent with the previous research [26, 61–66]. The unique hierarchical micro-mesoporous shell of CANHCSs with large macroporous cores is advantageous for improving the catalytic activity because of enhanced mass-transfer kinetics and the accessibility of active sites in mesopores, accumulated electrolyte ions in micropores and large storage capacity of the electrolyte in macroporous cores [67].

**Fig. 3:**
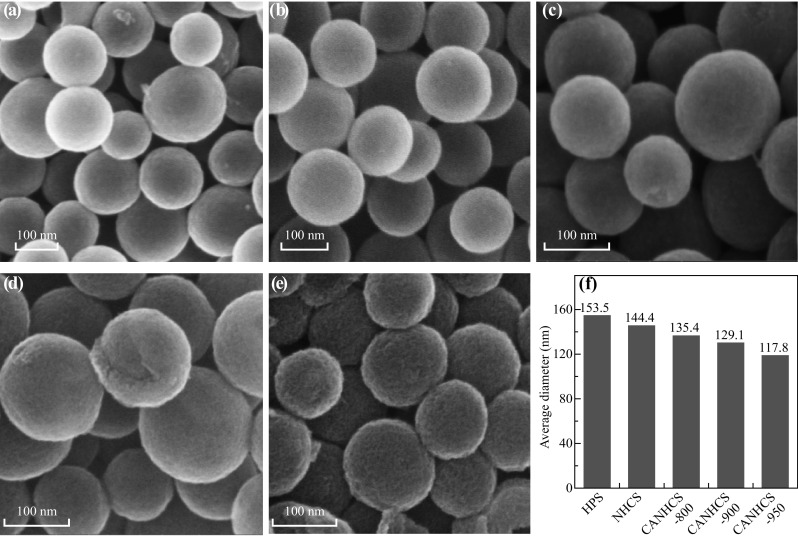
SEM images of **a** HPSs, **b** NHCSs, **c** CANHCS-800, **d** CANHCS-900, **e** CANHCS-950 and **f** average diameters of carbon spheres calculated from panel **a**–**e**

The effects of CO_2_ activation on the morphologies of carbon spheres were studied by SEM and TEM. As shown in Fig. 3, the as-prepared HPSs consist of well-dispersed spherical particles (Fig. 3a), and their morphology is still well retained after treatment in NH_3_ flow (Fig. 3b). Subjected to the CO_2_ activation treatment, CANHCSs still maintain excellent spherical morphology, but their surfaces evidently become rougher and rougher with increasing the temperature (Fig. 3c–e), implying the activation process has a great effect on the surface of spheres. Specifically, in spite of 90% carbon loss at 950 °C, the resulting CANHCS-950 still maintains the parent morphology of NHCSs. However, the activation treatment under CO_2_ atmosphere leads to the evident decrease in the size of the particles, as evidenced by size distributions (Fig. S1). The original NHCSs have the diameters of 100–165 nm and an average diameter of 144.4 nm. As shown in Fig. 3f, after the activation with CO_2_, the sizes of the CANHCSs are evidently smaller than that for NHCSs, and their sizes present the decreasing trend with increasing the activation temperature, well matching with the trend of carbon yields. The CANHCS-800, CANHCS-900 and CANHCS-950 have an average diameter of 135.4, 129.1 and 117.8 nm, respectively, significantly smaller than that for NHCSs, which could be mainly ascribed to the following two issues: (1) the carbon framework exposed to the higher temperatures generally resulted in the contraction of the lattice and reduction in the pore sizes and (2) etching off more carbon with CO_2_ at higher temperature also could cause the decrease in the sizes of carbon spheres.

**Fig. 4:**
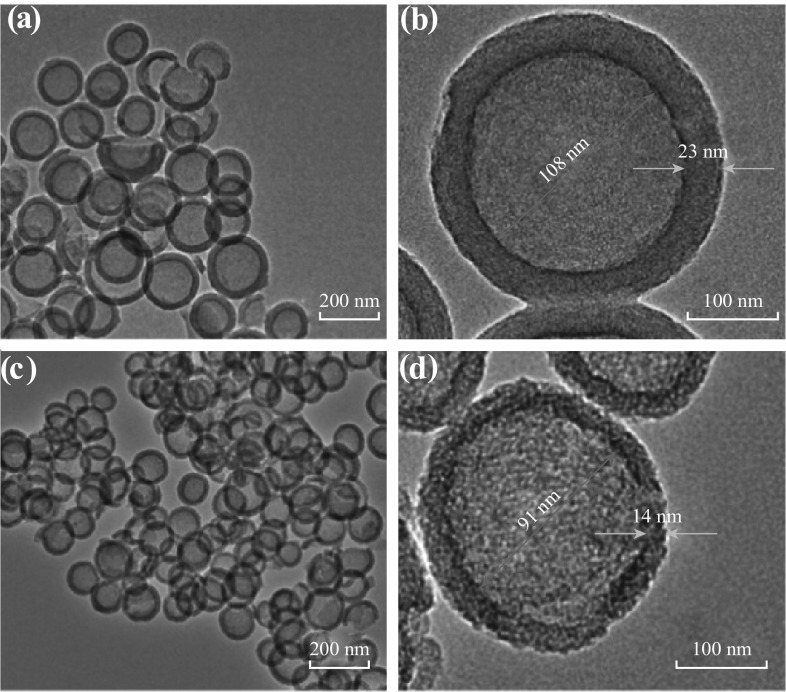
TEM images of **a**–**b** NHCSs and **c**–**d** CANHCS-950

As shown in Fig. 4a, b, it is clearly observed that NHCSs have a hollow spherical structure with the shell thickness of ~23 nm and hollow core of ~108 nm. After the activation with CO_2_ at 950 °C, the sizes of the resulting CANHCS-950 evidently decreased (Fig. 4c), but the spherical morphology is still kept well (Fig. 4d), matching with the SEM observation. The higher-resolution TEM image of a single sphere for CANHCS-950 shows that CO_2_ activation has a great effect on the microstructure of carbon sphere. It has a smaller thickness of ~14 nm and hollow core of ~91 nm than those for NHCSs (Fig. 4d). The activated spheres look like much looser than that in Fig. 4b, and many white fine “dots” are clearly observed, which should be the pores formed by etching off carbon with CO_2_. More information about pore textures could be obtained from the N_2_ sorption measurements.

**Fig. 5:**
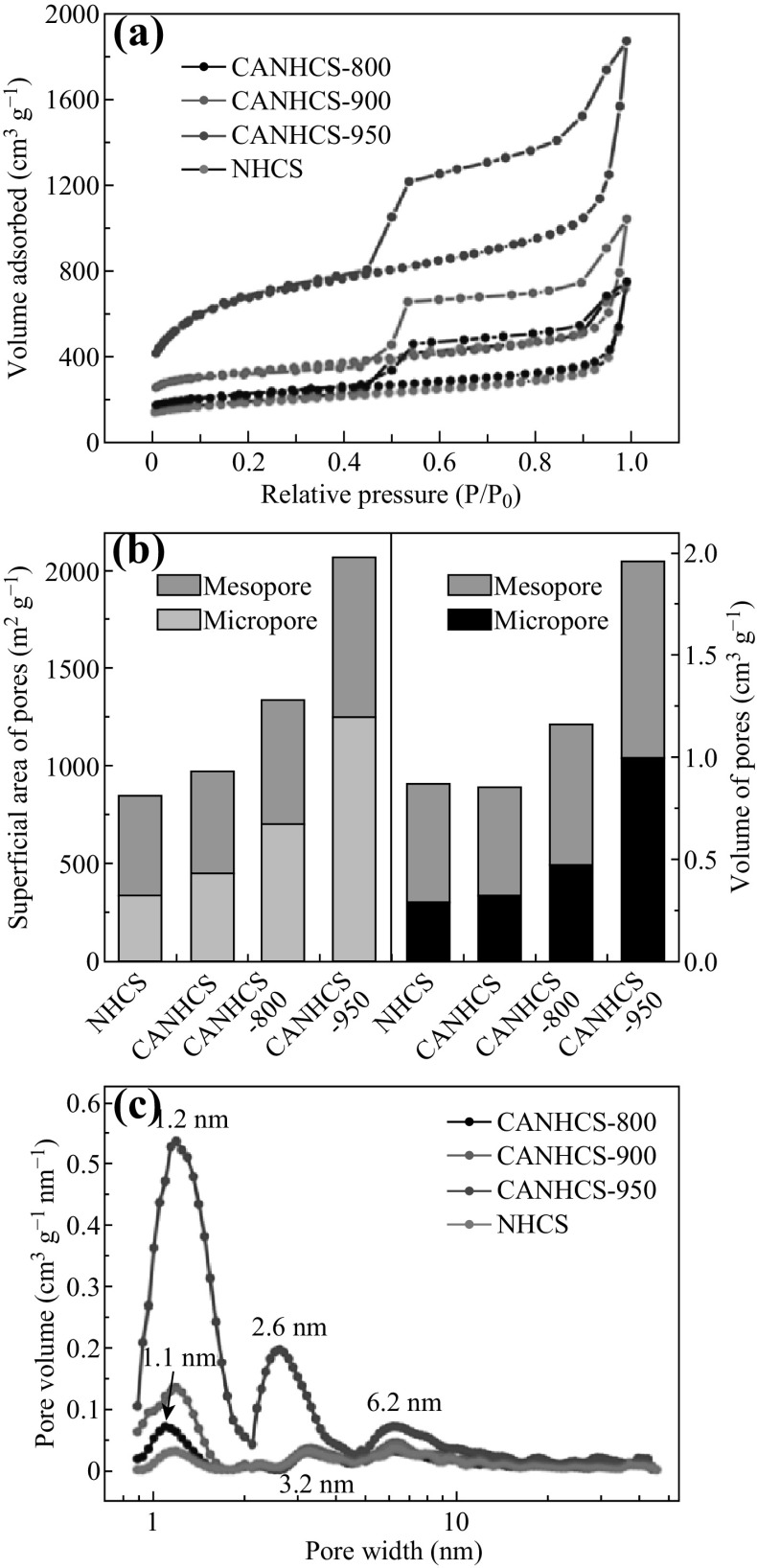
**a** N_2_ adsorption–desorption isotherms, **b** the superficial areas and pore volumes in the range of micropore and mesopore, **c** pore distribution curves of NHCSs, CANHCS-800, CANHCS-900, and CANHCS-950

All the pore characteristics of the samples were tested by the N_2_ adsorption–desorption experiments at −196 °C. The results of pore structures of the samples are summarized in Table 1. The surface areas were determined by the BET method while the surface area of micropores and mesopores were determined by the t-plot method. The values of total volume and micropore volume were calculated according to the DFT method and HK method, respectively. As shown in Fig. 5a, the N_2_ adsorption–desorption isotherms of all samples are typical type IV with a H1-type hysteresis loop. There is a sharp capillary condensation step in the relative pressure range from 0.40 to 0.55. It means that these materials have both mesopores and micropores. NHCSs have a surface area of 846 m^2^ g^−1^ in which the micropore surface (331 m^2^ g^−1^) dominates 39.13%. The adsorbed amount evidently increases with the increased activation temperature (Fig. 5a), indicating the larger surface areas for the activated samples. Indeed, CANHCS-800, CANHCS-900 and CANHCS-950 have a specific surface area value of 972, 1338, and 2072 m^2^ g^−1^, respectively. This trend is opposite to that for their mass losses (Fig. 2), implying these pores were formed by etching with CO_2_. It is notable that the percent of micropore areas significantly increases (from 39.13% for NHCSs to 46.19% for CANHCS-800, 52.47% for CANHCS-800 and 60.33% for CANHCS-800) with higher activation temperatures and indicates that the CO_2_ activation is beneficial for micropore formation. Similarly, the micropore volumes and the percent of micropore volumes also increase as the activation temperature increases. Although the percent of mesopore areas and volumes for the activated samples decreased, their actual surface areas and pore volumes evidently increase with the increased activation temperatures (Fig. 5b). Anyway, CANHCS-950 has the largest surface area of 2072 m^2^ g^−1^ and pore volume of 1.96 cm^3^ g^−1^ among these samples. Moreover, the ultra-large surface area and pore volume are expected to show the improved ORR activity since the porous materials with well-defined porosity, high specific surface area and pore volume not only have more accessible active sites, but also promote the efficient transport of the electrolyte, reactants and products [68].

**Table 1:** Texture parameters of NHCSs and CANHCSs activated at different temperatures

Samples	*S*_BFT_^a^ (m^2^ g^−1^)	*S*_micro_^b^ (m^3^ g^−1^)	*S*_micro_/*S*_BET_ (%)	*V*_total_^c^ (cm^3^ g^−1^)	*V*_micro_^d^ (cm^3^ g^−1^)	*V*_micro_/*V*_total_ (%)	Pore size (nm)^e^	
							Micro	Meso
NHCSs	846	331	39.13	0.87	0.29	33.33	1.2	3.2/6.2
CANHCS-800	972	448	46.09	0.86	0.32	37.21	1.1	3.2/6.2
CANHCS-900	1338	702	52.47	1.16	0.48	41.38	1.2	3.3/6.2
CANHCS-950	2072	1250	60.33	1.96	0.99	50.51	1.2	2.6/6.2

^a^Surface area determined by the BET method

^b^Micropore surface area determined by the t-plot method

^c^DFT method cumulative pore volume

^d^Micropore volume by the HK method

^e^Maximum values of the pore size distribution determined by the DFT method

The pore size distributions for NHCSs and CANHCSs are presented in Fig. 5c. The original NHCSs show three maximum values (1.2, 3.2, and 6.2 nm) of pore sizes (Table 1). Upon activation, the volume adsorbed improves obviously with the increment of the activation temperature (Fig. 5c). However, the higher activation temperature significantly leads to the widening of pores, although the maximum position value for mesopore size (2.6 nm) changes smaller. Such a hierarchical micro-mesoporous N-doped carbon shells with large hollow macroporous cores could contribute to the efficient storage of electrolyte in macroporous cores, fast transport of the electrolyte, reactants and products through mesopores, and effective accumulation of electrolyte ions in micropores, thus improving the electrocatalytic ORR performance.

**Fig. 6:**
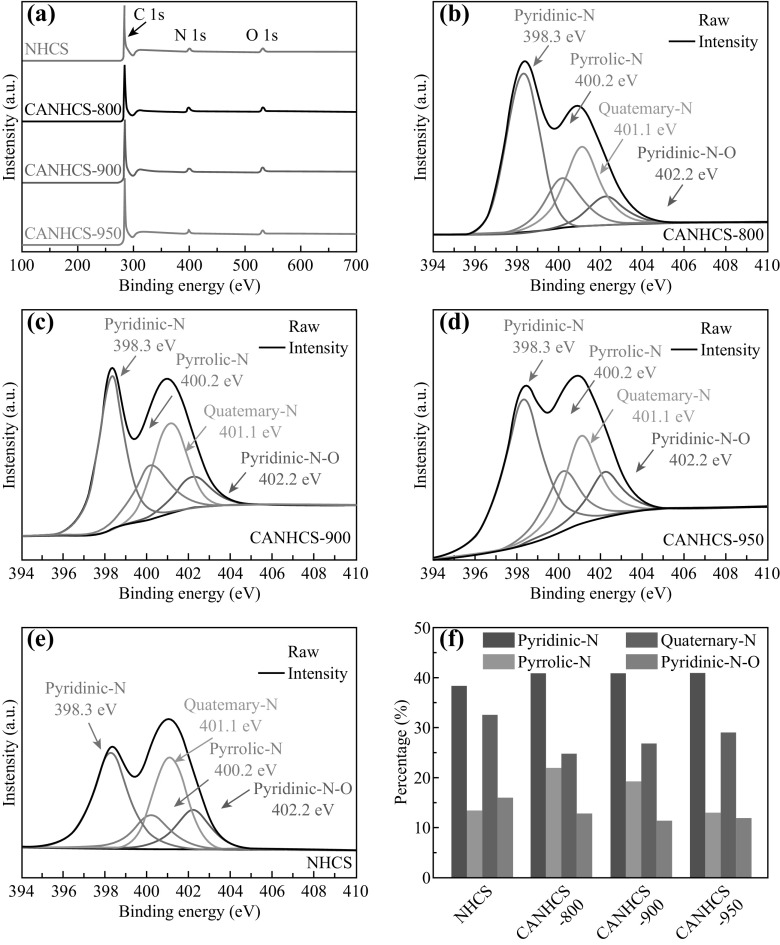
**a** XPS spectra of NHCSs, CANHCS-800, CANHCS-900 and CANHCS-950, **b**–**e** high-resolution XPS analysis of N 1s for **b** CANHCS-800, **c** CANHCS-900, **d** CANHCS-950 and **e** NHCSs. **f** relative atomic percentage (%) of different N functionalities received from the N 1s peaks

The effect of CO_2_ activation on the functional groups of the surfaces’ nature could be obtained by XPS. XPS survey spectra and high-resolution N1s spectra for all samples are shown in Fig. 6a–e. In Fig. 6a, there are obvious signals from carbon (C1s, ~284.8 eV), nitrogen (N1s, ~398.4 eV) and oxygen elements (O1s, ~532.2 eV) for all samples. The summaries of elemental compositions of CANHCSs are listed in Table 2. The presence of the N1s peak in the spectrum of NHCSs indicates that the N atoms have been successfully doped within the carbon framework by pyrolysis in NH_3_ (Fig. 6a). For the CANHCSs, the contents of carbon increase gradually while those of nitrogen drop off from 7.6 to 3.8 at% with the activation temperature rising from 800 to 950 °C. Therefore, the N/C molar ratios for CANHCSs decrease with increasing the activation temperature, suggesting the elimination of N atoms at higher temperatures. It is known that CO_2_ can react with carbon materials under high temperature to form plenty of micropores and narrow mesopores, thus resulting in the decrease in the nitrogen contents. The doping of N atoms into carbon could enhance the electrocatalytic activity because N doping can modify the electronic structures, chemical activities and Fermi level of the adjacent carbon atoms, favorably adsorbing and activating O_2_ molecules. However, the total nitrogen content is a less important factor affecting the activity than the nitrogen binding configurations and their relative contents [69]. Thus, the detailed nitrogen bonding configurations were further studied by high-resolution N1s XPS spectra.

**Table 2:** Elemental compositions of CANHCSs determined by the XPS analysis

Samples	Carbon (at%)	Nitrogen (at%)	Oxygen (at%)	N/C molar ratio
CANHCS-800	88.5	7.6	3.9	0.086
CANHCS-900	90.0	5.5	4.5	0.061
CANHCS-950	91.8	3.8	4.4	0.041

As shown in the high-resolution XPS analysis of N1s spectra (Fig. 6b–e), four kinds of nitrogen functional groups are doped including pyridinic N, pyrrolic N, quaternary N and pyridinic N–O which center at ~398.3, ~400.2, ~401.1, and ~402.2 eV for binding energy, respectively [36, 50, 70]. The peak positions and relative compositions of different N groups of trained from the N1s signals are shown in Fig. 6f and Table 3. Obviously, the nitrogen species of the samples vary depending on the activation temperature. It has been reported that both pyridinic and graphitic N groups could promote the electrocatalytic ORR by lowering the overpotential and increasing the current density. Based on the peak areas, it is found that the pyridinic and graphitic groups dominates most of the N groups in these CANHCSs, and the relative content of pyridinic N groups is more than 40 at%. Specifically, CANHCS-950 possesses the largest relative contents of pyridinic and graphitic N groups. Both pyridinic and graphitic N groups are considered as the active sites for the ORR, which could lower the overpotential and increase the current density [30, 71, 72]. Considering its highest surface area and unique hierarchical structure with hollow core, it is expected that CANHCS-950 could demonstrate the superior ORR activity.

**Table 3:** Peak positions and relative compositions of different N functional groups obtained from the high-resolution N1s signals

Sample	Pyridine N		Pyrrolic N		Quaternary N		Pyridinic N–O	
	Position (eV)	at%	Position (eV)	at%	Position (eV)	at%	Position (eV)	at%
NHCSs	398.3	38.2	400.2	13.4	401.1	32.4	402.2	16.0
CANHCS-800	398.3	40.9	400.2	21.7	401.1	24.7	402.2	12.7
CANHCS-900	398.3	42.9	400.2	19.2	401.1	26.7	402.2	11.2
CANHCS-950	398.3	46.5	400.2	12.8	401.1	28.9	402.2	11.8

**Fig. 7:**
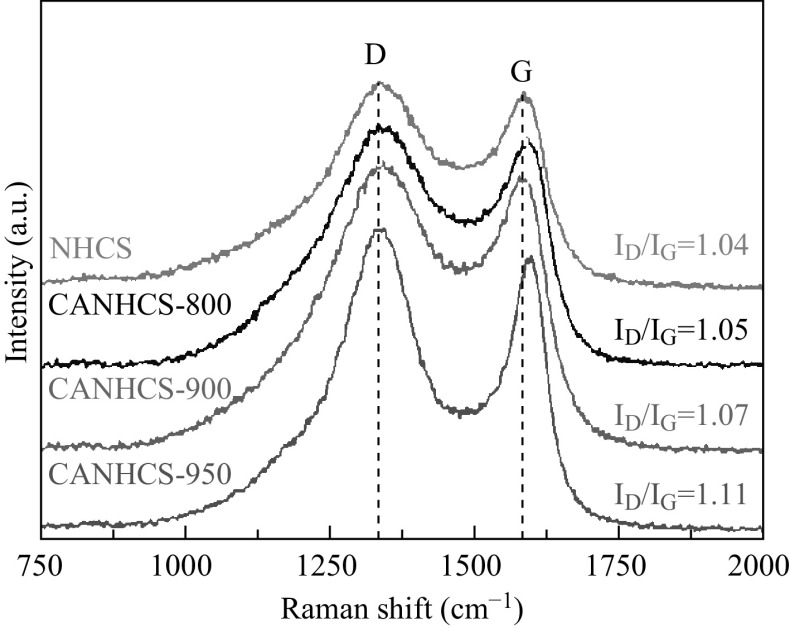
Raman spectra of NHCSs, CANHCS-800, CANHCS-900 and CANHCS-950

The structural information and the existence of the graphitic domains on the samples are further obtained by Raman spectroscopy (Fig. 7). The disorder-induced D-band is ranging from 1200 to 1450 cm^−1^, and the tangential stretch G band is between 1500 and 1600 cm^−1^ [73]. There is the disordered carbon peak “D” at ~1330 cm^−1^ and the graphitic carbon peak “G” at ~1580 cm^−1^. Both peaks of CANHCSs become stronger as the activation temperature goes on, and all of them are higher than those for NHCSs. The D peak refers to the structural defects on the graphitic plane and partially disordered structures in carbon materials, whereas the G peak corresponds to the *E*_2g_ vibration of graphitic carbon skeleton [74]. The degree of graphitization, defects and edge sites can be found by analyzing the relative intensities of these two lines depend on the type of graphitic materials [75]. The specific value of D peak and G peak can be used to indicate the graphitization degree. The values *I*_D_/*I*_G_ ratios increase from 1.04 for NHCSs to 1.05 for CANHCS-800, 1.07 for CANHCS-900 and 1.11 for CANHCS-950, indicating more defects and edge sites were created by CO_2_ activation.

The dimension of graphite crystallite *L*_a_ which formed the amorphous carbon can be figured out by Eq. 4 and was inversely proportional to *I*_D_/*I*_G_ [76]. The relationship of the parameters is as follows:

4 $$\frac{{I_{\text{D}} }}{{I_{\text{G}} }} = \frac{{C\left( {\lambda_{\text{L}} } \right)}}{{L_{\text{a}} }}$$

Therein, *λ*_L_ is the wavelength of incident light and the *C*(*λ*_L_) is a coefficient about *λ*_L_. In the event that the *λ*_L_ is between 400 and 700 nm, the *C*(*λ*_L_) is determined by Eq. 5 [77]:

5 $$C\left( {\lambda_{L} } \right) \, \approx \, 0.33 \times \lambda_{L} - 12.6$$

On the basis of Eqs. 4 and 5, the value of *L*_a_ decreases along with increase in *I*_D_/*I*_G_. It means that the dimension of graphite crystallite changes thinner and the graphitization degree for the activated samples is lower than that for NHCSs. At high temperature, CO_2_ can react with carbon locating on the edge of crystallite to form CO, thus bringing massive micropores and vacancies on the surface of carbon spheres.

**Fig. 8:**
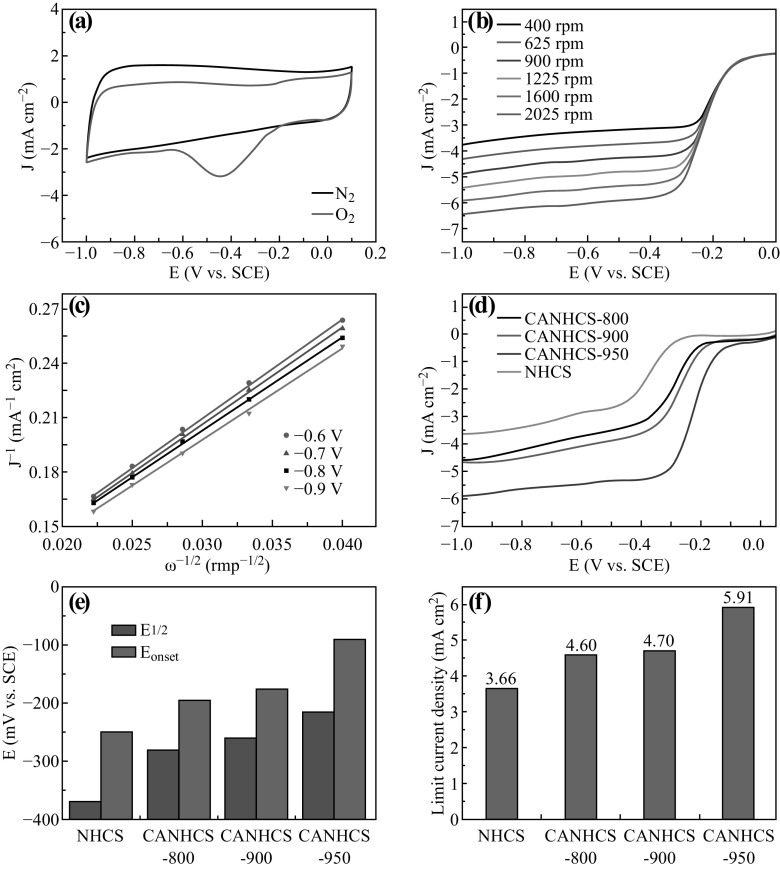
**a** Cyclic voltammograms (CVs) of CANHCS-950 in N_2_ and O_2_-saturated 0.1 M KOH electrolyte at a scan rate of 50 mV s^−1^. **b** Linear sweep voltammetry (LSV) curves of CANHCS-950 in O_2_-saturated 0.1 M KOH electrolyte at different rotation rates from 400 to 2025 rpm. **c** K-L plots (i^−1^ vs. ω^−1/2^) for CANHCS-950 calculated from LSV at diffident electrode potentials. **d** LSV curves of CANHCS-800, CANHCS-900, CANHCS-950 and NHCSs in O_2_-saturated 0.1 M KOH solution at a scan rate of 10 mv s^−1^ and 1600 rpm. **e** The onset potential (*E*_onset_) and the half-wave (*E*_1/2_) of CANHCS-800, CANHCS-900, CANHCS-950 and NHCSs in O_2_-saturated 0.1 M KOH solution at a scan rate of 10 mV s^−1^ and 1600 rpm. **f** Limiting current density of CANHCSs and NHCSs in O_2_-saturated 0.1 M KOH at a scan rate of 10 mV s^−1^ and 1600 rpm

The ORR activities of the samples were investigated by measuring the cyclic voltammograms (CV) and rotating disk electrode (RDE) voltammograms in 0.1 M O_2_-saturated KOH solution using a conventional three-electrode system. As shown in Fig. S2, the CV curves show that the cathodic peak of CANHCS-950 is more positive than those for other samples. The parameters of peak positions are listed in Fig. S2b. The ORR peaks at −0.31, −0.29, and −0.27 V (vs. SCE) could be found for CANHCS-800, CANHCS-900 and CANHCS-950, respectively. For comparison, un-activated NHCSs was also measured and its ORR peak position is −0.39 V (vs. SCE). Figure 8a shows that the CV curve exhibits a pronounced peak in O_2_-saturated electrolyte at −0.27 V (vs. SCE) while no cathodic peak is observed in N_2_-saturated 0.1 M KOH solution. It implies that CANHCS-950 catalyst possesses the real ORR activity.

Further measurements using the RDE were carried out by linear sweep voltammetry (LSV) in O_2_-saturated 0.1 M KOH at a rotating speed of 400–2025 rpm (sweep rate: 10 mV s^−1^). The results of LSV for CANHCS-950, CANHCS-800 and CANHCS-900 are shown in Fig. 8b and Fig. S3a, b, respectively. The current densities of the LSV curves steadily increased with the increase in the rotating speed, implying mass transport emerges on the electrode surface. The kinetic parameters could be analyzed with the following Koutecky–Levich (K-L) Eqs.:

6 $$\frac{1}{J} = \frac{1}{{J_{\text{k}} }} + \frac{1}{{B\omega^{1/2} }}$$

7 $${\text{B}} = 0.2{\text{nFC}}_{{{\text{O}}_{2} }} {\text{D}}_{{{\text{O}}_{2} }}^{2/3} \nu^{ - 1/6}$$

where *J* is the measured current density, *J*_k_ is the kinetic limiting current density, *ω* is the electrode rotating rate, *n* is the electron transfer number, F is the Faraday constant (F = 96,485 C mol^−1^), C_O2_ is the bulk concentration of O_2_ for 0.1 M KOH (C_O2_ = 1.2 × 10^−6^ mol cm^−3^), *D*_O2_ is the diffusion coefficient of O_2_ for 0.1 M KOH (D_O2_ = 1.9 × 10^−5^ cm^2^ s^−1^) and *v* is the kinetic viscosity (*v* = 0.01 cm^2^ s^−1^).

The K-L plots (*j* ^−1^ vs. *ω* ^−1/2^) are shown in Fig. 8c, which is drawn with the values of currents at different voltage ranging from −0.6 to −0.9 V (vs. SCE) and various rotation speeds. It can be seen that the dates exhibit great linearity, running parallel with each other. In order to compare the electrocatalytic activities of the samples, the LSV measurements in O_2_-saturated 0.1 M KOH at a rotating speed of 1600 rpm for CANHCSs and NHCSs are listed in Fig. 8d. All CANHCSs possess more positive onset (*E*_onset_) and half-wave (*E*_1/2_) potential, and larger limiting current density than NHCSs (Fig. 8d–f), implying CO_2_ activation has a significant positive effect on the ORR activity in spite of the evident N loss, possibly due to enhanced specific surface area and hierarchical microstructure. It has been suggested that the large surface area could play more important role in improving the ORR activity by increasing the mass transport. Among three CANHCSs, CANHCS-950 shows the best ORR activity, reflected by its most positive *E*_onset_ (−89 mV vs. SCE) and *E*_1/2_ and largest limiting current density (5.91 mA cm^−2^). This current density is much superior to those previously reported for various N-doped carbons materials including N-graphene [14], N-carbon spheres [77] and N-CNT/G [52, 78]. The electrochemical ORR performances of various N-doped carbon shells are listed in Table S1 for comparison. Relative to NHCSs and other CANHCSs, the improvement of CANHCS-950 on the ORR activity was ascribed mainly to its largest surface area and highest relative contents of pyridinic and graphitic N groups, which favorably promote the fast transport/diffusion of reactants, ions and electrons to exposed active sites.

**Fig. 9:**
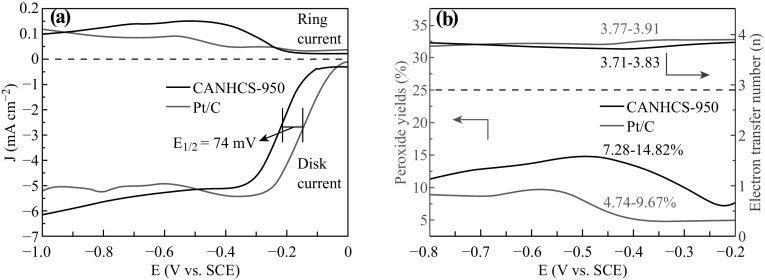
**a** Rotating ring-disk electrode (RRDE) voltammograms for CANHCS-950 and Pt/C in O_2_-saturated 0.1 M KOH electrolyte at a rotation rate of 1600 rpm with a ring potential at 0.2 V (vs. SCE) and **b** number of electrons transferred and peroxide yields of CANHCS-950 and Pt/C in O_2_-saturated 0.1 M KOH electrolyte

In order to further discuss the electrochemical performance and the detailed investigation of the mass-transfer kinetics of CANHCS-950, a rotating ring-disk electrode (RRDE) measurement in O_2_-saturated 0.1 M KOH electrolytes at a rotation rate of 1600 rpm was tested. For comparison, 20 wt% Pt/C was also tested. As shown in Fig. 9a, the gap of *E*_1/2_ between CANHCS-950 and Pt/C is about 74 mV, evidently smaller than those reported for various N-doped carbons including NCNTs [78] and N–carbon nanocages [79]. The electron transfer number (*n*) and peroxide yields are calculated from the RRDE data over the potential range from −0.8 to −0.3 V (vs. SCE) according to Eqs. 3 and 4. Figure 9b presents that the electron transfer number (*n*) of CANHCS-950 varies in the range of 3.71–3.84, very close to that for Pt/C. It implies that the catalytic ORR process of CANHCS-950 is a 4e^−^-dominant reaction pathway. Note that four-electron process is very important for ORR in fuel cells, because peroxides can poison the cell. Indeed, the peroxide yields for the ORR by CANHCS-950 was calculated to be 7.28%–14.82% while the values of Pt/C range from 7.47% to 9.67%, implying a highly desirable ORR process with low yield of peroxide for CANHCS-950 to obtain maximum energy capacity. Thus, CANHCS-950 with unique hierarchical structures and large surface area could be regarded as an excellent candidate as metal-free electrocatalysts toward the ORR.

**Fig. 10:**
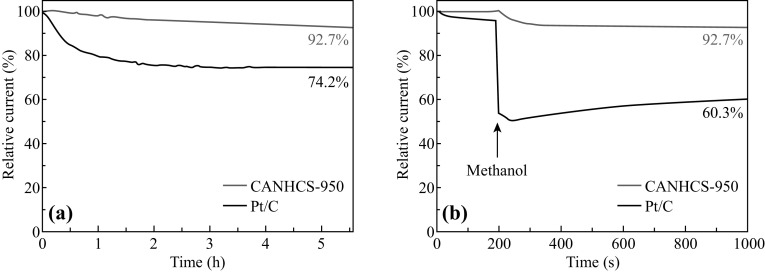
**a** Current–time (i-t) chronoamperometric responses for CANHCS-950 and 20% Pt/C in O_2_-saturated 0.1 M KOH electrolyte at −0.6 V (vs. SCE) for the ORR at a rotating speed of 1600 rpm and **b** comparison of chronoamperometric responses over 1000 s at a constant rotation speed of 1600 rpm for CANHCS-950 and Pt/C in O_2_-saturated 0.1 M KOH electrolyte with 3 M methanol added at 200 s

Stability is of great importance for catalysts in practical applications in fuel cell technology. The stability was tested by the chronoamperometric measurements at −0.6 V (vs. SCE) in O_2_-saturated 0.1 M KOH electrolyte at a rotating speed of 1600 rpm. Pt/C was also measured as a control sample to check whether CANHCS-950 has better stability under the same environment. As shown in Fig. 10a, after 5.5 h of continuous operation, there is an apparent decrease in the current density for Pt/C electrode. The current density retention rate of Pt/C electrode is only about 74.2% while the CANHCS-950 catalyst still retains 92.7% of the initial current density. Namely, the CANHCS-950 catalyst has the better stability for the ORR than Pt/C. The methanol poisoning effect of catalyst is another issue that needs to be addressed in practical applications in fuel cell technology in direct methanol fuel cells (DMFC). To analyze this issue, samples were measured by chronoamperometric measurements at −0.6 V (vs. SCE) in O_2_-saturated 0.1 M KOH electrolyte at a rotating speed of 1600 rpm for 1000 s while 3 M methanol was added at 200 s. As shown in Fig. 10b, after the injection of methanol, the Pt/C electrode (black curve) decreases instantaneously for the voltammetric current and the current maintains about 60.3%. However, there is a slight change for CANHCS-950 and the retention rate keeps at 92.7% in the same process, which is much higher than that of Pt/C. These results indicate that CANHCS-950 can not only keep excellent stability in alkaline solution but also perform insensitive to methanol.

## Conclusions

In summary, a kind of novel N-doped carbon shells with hollow core and triple hierarchical micro-meso-macroporosity has been successfully prepared by the activation of N-doped hollow carbon spheres in CO_2_ atmosphere at high temperature. The surface areas, total pore volumes and micropore percentages of the CO_2_-activated samples evidently increase with increasing activation temperature from 800 to 950 °C, while the N contents show a contrary trend from 7.6 to 3.8 at% determined by XPS. The CANHCS-950 has a very high specific surface area of 2072 m^2^ g^−1^, pore volume of 1.96 cm^3^ g^−1^ and N doping of 3.8 at%. The pyridinic and graphitic N groups are dominant in the activated samples, which are regarded as the active sites for the ORR. Combined with their hierarchical structures, the CANHCSs are beneficial for storing the electrolyte in macroporous core and transfer the reactants and electrolyte to the active sites in the mesopores, thus improving the ORR activity. The CO_2_ activation treatment evidently improves the limiting current density and decrease the ORR onset potential. Among the activated samples, CANHCS-950 showed the comparable ORR activity but superior methanol tolerance and long-term durability to 20 wt% Pt/C with a 4e^−^-dominant transfer pathway in alkaline media. Such N-doped carbon materials with unique hierarchical structure and large surface area are also potential in many fields, such as adsorption of heavy metal ions, CO_2_ capture, gas storage and supercapacitors.

## Electronic supplementary material

Below is the link to the electronic supplementary material.

Supplementary material 1 (PDF 576 kb)
