# Supplementary material for: Creation of Triple Hierarchical Micro-Meso-Macroporous N-doped Carbon Shells with Hollow Cores Toward the Electrocatalytic Oxygen Reduction Reaction
Source: Nanomicro Lett. 2017 Sep 27;10(1):3. doi: 10.1007/s40820-017-0157-1 (PMC6199056; doi:10.1007/s40820-017-0157-1)
Supplement: Supplementary file 1 — Supplementary material 1 (PDF 576 kb) [file 40820_2017_157_MOESM1_ESM.pdf]

Supporting Information for

Creation of Triple Hierarchical Micro-Meso-Macroporous N-doped Carbon Shells with Hollow Cores towards the Electrocatalytic Oxygen Reduction Reaction

Ruohao Xing[1, 2], Tingsheng Zhou[2], Yao Zhou[2], Ruguang Ma[2], Qian Liu[2, 3, *], Jun Luo[1, 3], Jiacheng Wang[2, 3, *]

[1]School of Materials Science and Engineering, Shanghai University, 99 Shangda Road, Shanghai 200444, People's Republic of China

[2]State Key Laboratory of High Performance Ceramics and Superfine Microstructure, Shanghai Institute of Ceramics, Chinese Academy of Sciences, 1295 Dingxi Road, Shanghai 200050, People's Republic of China

[3]Shanghai Institute of Materials Genome, Shanghai, People's Republic of China

*Corresponding authors.
Email: jiacheng.wang@mail.sic.ac.cn, qianliu@mail.sic.ac.cn

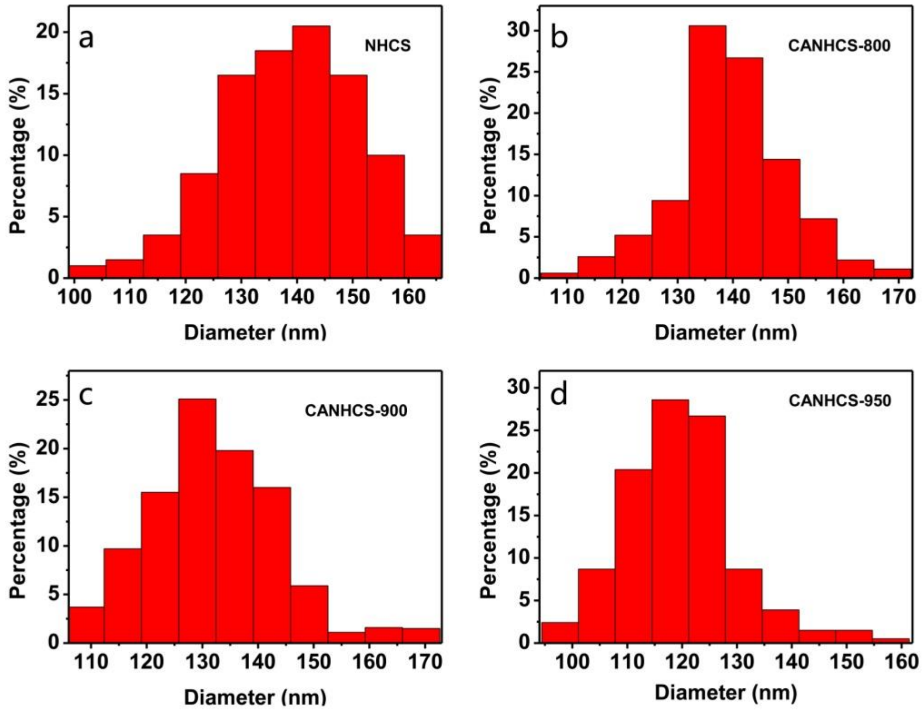

Fig. S1 Diameter distributions of **a** NHCS, **b** CANHCS-800, **c** CANHCS-900, **d** CANHCS-950

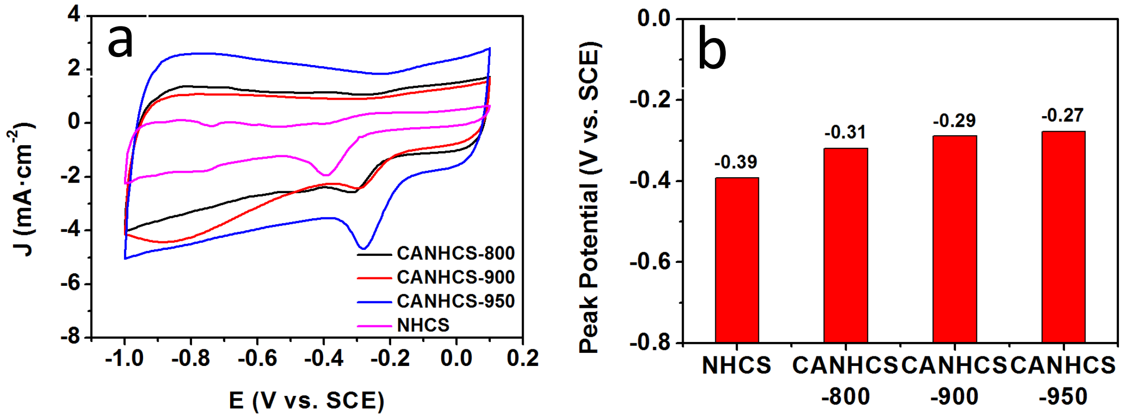

Fig. S2 **a** Cyclic voltammograms (CV) of the CANHCS-800, CANHCS-900, CANHCS-950, and NHCSs in O_2-saturated 0.1 M solution of KOH electrolyte at a scan rate of 50 mV s^{-1} and **b** the potential of ORR peak from the cyclic voltammograms (CV) in panel **a**

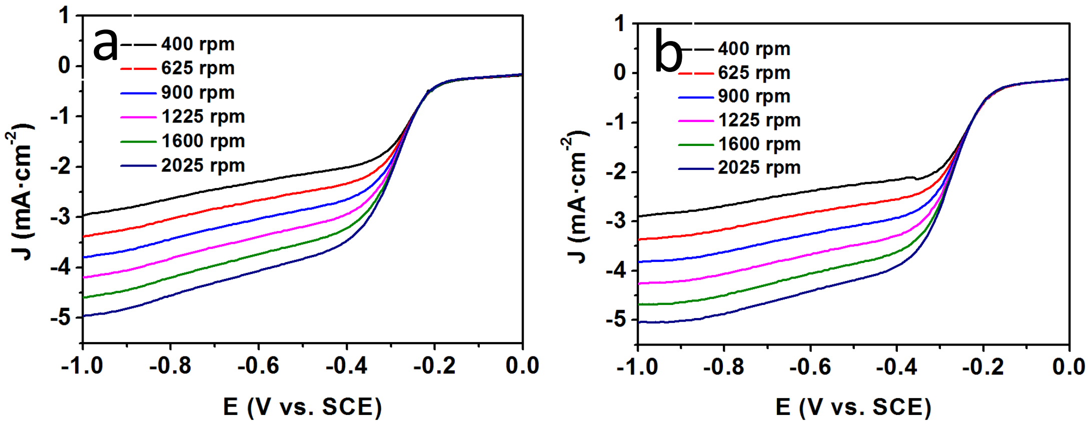

Fig. S3 Linear sweep voltammetry (LSV) curves of **a** CANHCS-800, **b** CANHCS-900 in O_2-saturated 0.1 M KOH electrolyte at different rotatingspeeds from 400 to 2025 rpm

Table S1 the comparison for the electrochemical performances of the N-doped carbon shells

Catalysts	E_{onset} - $E_{onset(Pt/C)}$ (mV vs. SCE)	$E_{1/2}$ – $E_{1/2(Pt/C)}$ (mV vs. SCE)	Current density (mA cm^{-2})	Ref.
CANHCS-950	70	74	5.91	This work
HMNC-0.5-800	60	100	4.25	[1]
N-CS	-5	0	3.2	[2]
HMC	50	0	5.0	[3]
NHCS	200	210	3.8	[4]
NHCS91	100	200	4.6	[5]
3D-960HGBs	150	110	4.9	[6]

References

[1] R. Wu, S. Chen, Y. Zhang, Y. Wang, Y. Nie, W. Ding, X. Qi, Z. Wei, Controlled synthesis of hollow micro/meso-pore nitrogen-doped carbon with tunable wall thickness and specific surface area as efficient electrocatalysts for oxygen reduction reaction. J. Mater. Chem. A **4**(7), 2433-2437 (2016). doi:10.1039/C5TA09859A

[2] G.A. Ferrero, K. Preuss, A.B. Fuertes, M. Sevilla, M.M. Titirici, The influence of pore size distribution on the oxygen reduction reaction performance in nitrogen doped carbon microspheres. J. Mater. Chem. A **4**(7), 2581-2589 (2016). doi:https://doi.org/10.1039/C5TA10063A

[3] L. Hadidi, E. Davari, M. Iqbal, T.K. Purkait, D.G. Ivey, J.G. Veinot, Spherical nitrogen-doped hollow mesoporous carbon as an efficient bifunctional electrocatalyst for zn-air batteries. Nanoscale **7**(48), 20547-20556 (2015). doi:https://doi.org/10.1039/C5NR06028A

[4] Z. Wu, R. Liu, J. Wang, J. Zhu, W. Xiao, C. Xuan, W. Lei, D. Wang, Nitrogen and sulfur co-doping of 3D hollow-structured carbon spheres as an efficient and stable metal free catalyst for the oxygen reduction reaction. Nanoscale **8**(45), 19086 (2016). doi:https://doi.org/10.1039/C6NR06817K

[5] J. Sanetuntikul, T. Hang, S. Shanmugam, Hollow nitrogen-doped carbon spheres as efficient and durable electrocatalysts for oxygen reduction. Chem. Commun. **50**(67), 9473-9476 (2014). doi:https://doi.org/10.1039/C4CC03437F

[6] Y. Lu, M. Liu, H. Nie, C. Gu, M. Liu, Z. Yang, K. Yang, X.A. Chen, S. Huang, Direct fabrication of metal-free hollow graphene balls with a self-supporting structure as efficient cathode catalysts of fuel cell. J. Nanoparticle Res. **18**(6), 1-9 (2016). doi:10.1007/s11051-016-3457-3
